# Context-Aware Pending Interest Table Management Scheme for NDN-Based VANETs

**DOI:** 10.3390/s22114189

**Published:** 2022-05-31

**Authors:** Waseeq Ul Islam Zafar, Muhammad Atif Ur Rehman, Farhana Jabeen, Sanaa Ghouzali, Zobia Rehman, Wadood Abdul

**Affiliations:** 1 Department of Computer Science, COMSATS University, Islamabad 45550, Pakistan; zobia.rehman@comsats.edu.pk; 2 Department of Computing and Mathematics, Manchester Metropolitan University, Manchester M15 6BH, UK; atif_r@outlook.com; 3 Department of Information Technology, College of Computer and Information Sciences, King Saud University, Riyadh 11543, Saudi Arabia; sghouzali@ksu.edu.sa; 4 Department of Computer Engineering, College of Computer and Information Sciences, King Saud University, Riyadh 11543, Saudi Arabia; aabdulwaheed@ksu.edu.sa

**Keywords:** NDN, context-aware naming, VANETs, NDN, PIT, safety content dissemination, Pending Information Table, non-safety content, Named Defined Networking (NDN)

## Abstract

In terms of delivery effectiveness, Vehicular Adhoc NETworks (VANETs) applications have multiple, possibly conflicting, and disparate needs (e.g., latency, reliability, and delivery priorities). Named Data Networking (NDN) has attracted the attention of the research community for effective content retrieval and dissemination in mobile environments such as VANETs. A vehicle in a VANET application is heavily reliant on information about the content, network, and application, which can be obtained from a variety of sources. The information gathered can be used as context to make better decisions. While it is difficult to obtain the necessary context information at the IP network layer, the emergence of NDN is changing the tide. The Pending Information Table (PIT) is an important player in NDN data retrieval. PIT size is the bottleneck due to the limited opportunities provided by current memory technologies. PIT overflow results in service disruptions as new Interest messages cannot be added to PIT. Adaptive, context-aware PIT entry management solutions must be introduced to NDN-based VANETs for effective content dissemination. In this context, our main contribution is a decentralised, context-aware PIT entry management (CPITEM) protocol. The simulation results show that the proposed CPITEM protocol achieves lower Interest Satisfaction Delay and effective PIT utilization based on context when compared to existing PIT entry replacement protocols.

## 1. Introduction

Rapid advancements in various network technologies, such as Wireless Local Area Networks (WLANs) and cellular systems promise to accelerate the advancement of Intelligent Transportation Systems (ITS) [1]. Many cities around the world are experiencing population growth and rapid urbanization, as well as an increase in vehicle traffic relative to road infrastructure. The number of deaths caused by traffic accidents is by far the highest of any category of accidental deaths every year. ITS introduced Vehicular Ad hoc NETwork (VANET) to create a safer architecture for road transportation in order to make the journey safer, less stressful, and more enjoyable [2]. In VANETs, each vehicle relies on the processing, storage, and communication capabilities of On-Board Units (OBUs), which manufacturers are already incorporating into vehicles. As VANET is a critical component of ITS, research academies and industrialists are paying close attention. The VANETs communication capabilities enable vehicles to communicate with one another and with infrastructure (such as a Road-Side Unit (RSU)). The capability of in-vehicle technology enables a wide range of applications in which vehicles interact and collaborate with one another and with infrastructure. VANET was created primarily to support safety-related applications. Recently, autonomous and coordinated driving applications have emerged as yet another compelling reason to adopt VANETs [3]. In addition, infotainment applications continue to entice drivers who want to exchange multimedia content while driving.

In traditional Internet Protocol (IP)-based VANETs, each vehicle must be identified with a unique address, similar to Mobile Ad hoc Networks (MANETs) [3]. The IP communication model is based on host-to-host communications, in which one host requests a resource and others provide it. VANET is an information-centric network, where in most applications the vehicles care about information. Named Data Networking (NDN), which is one of the realizations of ICN, decouples content from producers and retrieves content from the nearest content holder using hierarchically and semantically meaningful names [4,5]. The closest content holder could be the original content producer or a node with a valid copy of the requested content. By increasing the number of content sources, NDN reduces content delivery delay and thus increases content delivery probability.

The NDN architecture is a promising replacement for the TCP/IP architecture [4,5]. As mentioned before, NDN is a content-centric protocol that focuses on the desired data, not the location of the data. NDN architecture supports mobility. Consumer (content requester) mobility is by default supported in the original design of NDN. NDN allows for assigning unique names to contents that can be used for its search, retrieval, storage, etc. Moreover, NDN supports ubiquitous in-network distributed caching, which allows intermediate routers (vehicles) to cache content to reduce the delay in obtaining content. NDN supports two types of messages: (i) Interest messages and (ii) Data messages. Customers request content by sending out an Interest message. The content holder who has the requested content forwards the Data message after receiving the Interest message. Each node in the native NDN contains three data structures. The Forwarding Information Base (FIB) data structure stores forwarding information by keeping a mapping of content name prefixes and interfaces (Faces) through which Data messages can be forwarded. The Pending Interest Table (PIT) keeps track of all pending Interests and their incoming interfaces that have yet to be served. The content objects are cached by the Content Store (CS) based on a caching policy. In NDN-based VANETs, content is requested by name. Additional information about the requested content may be included in the name.

Various VANET applications have multiple, possibly conflicting, and disparate QoS expectations [5,6,7,8,9]. For example, safety applications have low latency requirements, whereas non-safety applications do not have low latency requirements. Inefficient safety-related content dissemination could result in fatalities and disabilities. In the event of a road hazard, information must be received at the nearest traffic police station as soon as possible. Otherwise, it may cause traffic congestion, resulting in injuries, property damage, and lost time for motorists and passengers. In VANET applications, a vehicle is highly dependent on information about the content, network, and application, which can be obtained from a variety of sources [8,9]. The information gathered can be used as context to make better decisions [8,9]. In the case of VANETs, the context may include node position, speed, time when the content is generated, location where the content is generated, content type (safety, non-safety), application to which the content belongs, content format, application popularity, neighbourhood conditions, and many others. Recently, NDN has gained increasing attention for content distribution in VANETs [4,5,6]. Taking into consideration the VANET characteristics, wireless environment, and applications, efficient and effective content dissemination in NDN-based VANETs is a well-known problem and faces unique challenges [5,6] including content naming, forwarding, and PIT management. Context information can be used to not only improve the performance of routing (forwarding) protocols, but can also be exploited in making dynamic decisions such as those related to selecting the appropriate forwarding protocol and making NDN’s pending interest table (PIT) management decisions. Context information (embedded in Interest messages, content names, and collected by vehicles) can be used to enable efficient and effective content distribution in NDN-based VANETs [5,8,9]. While it is difficult to obtain the necessary context information at the IP network layer, the emergence of NDN is changing the tide.

The Pending Information Table (PIT) is a key player in finding content in NDN. Due to the limited opportunities offered by current memory technologies, PIT size is a bottleneck. The stored PIT Entry (PITE) is removed either when the PIT Entry Lifetime (PEL) expires or the vehicle with PITE receives the required Data packet. Flooding of Interest packets in NDN-based VANETs can quickly increase PIT size. In VANETs, Interest flooding and a large PEL can exhaust the PIT, impacting the overall Interest Satisfaction Rate (ISR). PIT overflow result in service disruptions as new Interest messages PIT entries cannot be added to PIT. In case of overload, to make space for the future PIT entries, the current state-of-the-art method removes entries with low priority. Priority is computed based on the PEL and number of requests received for the content [10]. The existing state-of-the-art methods do not consider context such as application type or content type while computing the priority. Different delivery priorities may be necessitated for traffic of the same type. For example, the delivery of a video required for safe driving (to raise awareness about the area in bad weather) should take priority over the delivery of a cartoon film video to a child. The later video type is less critical.

In this work, therefore, we focus on PIT management. More specifically, the major contributions of this paper can be summarized as follows:

- In this work, we focus on the context-aware adaptive PIT management scheme named Context-aware PIT Entry Management (CPITEM). The CPITEM scheme supports efficient use of PIT, taking into the consideration that VANET applications may have different QoS needs. CPITEM exploits the context information collected by the vehicle and presents it in the Context-aware Content Name (CACN) [9]. CACN is one of the important information components of the Interest and Data packet header.
- A simulation-based experimental study is conducted in an NS-3-based NDN simulator (ndnSIM) [11] to check the performance of the proposed scheme with relevant and state-of-the-art scheme [10].

The remainder of the paper is organized as follows. Section 2 describes the applications of VANETs; Section 3 presents the background related to PIT management in NDN-based VANETs. Section 4 presents the context-aware PIT management mechanism for NDN-based VANETs; Section 5 presents the performance analysis; and finally, Section 6 concludes our work.

## 2. VANETs Applications

Typical VANETs applications can be broadly classified into two categories: (i) safety, (ii) non-safety applications.

### 2.1. Safety Applications

Applications under the safety subgroup include time-sensitive applications. If the relevant information is not delivered in time, its usefulness is lost. The quality of service demanded by safety applications is close to real-time. Inefficient safety-related content dissemination could lead to life loss and disabilities. Road safety applications primarily assist drivers in avoiding vehicle collisions and lowering crash fatality ratios [5,6,8,9]. The majority of applications in this category are location- and time-based. These applications have a more localised spatial scope in terms of the extent of a geographical area in which the information is required and considered valuable (referred to as spatial validity of the content) [5,6,9]. Some of the safety applications are as follows:

- Post-crash notification: The spatial validity of this application is 500 m, and the temporal validity is 30 s [5,6,9].
- Emergency vehicle warning: The spatial validity of this application is 500 m, and the temporal validity is 10 m [5,6,9].
- Dangerous road warning: The spatial validity of this application is 100 m, and the temporal validity is 10 s [5,6,9].

### 2.2. Non-Safety Applications

Comfort and entertainment applications are called non-safety applications that aim to improve the comfort levels of drivers and passengers and make travel more pleasant. [5,6,9]. Communication usually takes place between vehicles or between vehicles and an RSU. Non-safety applications can tolerate delays because they do not have strict real-time requirements. Vehicles can use spatial- and time-validity information to determine PEL, where to cache and whether to participate in message dissemination.

Some of the non-safety applications are as follows:

- Traffic navigation map: The spatial validity of the traffic navigation map is 10 km, and the time validity is 30 min [5,6,9].
- Entertainment/Multimedia applications: Music downloads, file sharing, home control, and other interactive entertainment applications are examples of interactive entertainment applications. Information is not required by all vehicles, but rather on-demand based on user preferences.
- Commercial advertisement: The spatial validity of this application is 1.5 km, and the temporal validity is 1–10 days [5,6,9].

## 3. PIT Management in NDN-Based VANETs

The PIT data structure contains information about Interests currently pending, its outgoing interfaces from where the data are yet to be received and the incoming interface that has not yet been served. In other words, PIT is held accountable to store the content name and other important information components of the Interest, for which no Data message has been received yet. It also contains information about recently satisfied Interests. PIT Entry (PITE) is a set of InRecords and a collection of OutRecords. InRecord and OutRecord comprised a set of attributes such as Nonce, reference to the face, and the timestamp at which the Interest arrives [12]. PITE is identified by the Interest name. Interest is uniquely identified by combining two fields: name and Nonce. Nonce is a random number generated by the consumer vehicle and inserted in the Interest header. The InterestLifetime field in the Interest packet controls the expiration of the Inrecord. If InterestLifetime is not specified, the value 4 s is used as the default. If an Interest is not satisfied within the InterestLifetime period, the relevant PITE is deleted [12,13]. When InterestLifetime elapses after the last Interest packet arrives, an in-record expires. When all the InRecords expire, the PITE expires. If a PITE contains at least one unexpired InRecord, it is said to be pending. When InterestLifetime elapses after the last Interest packet is sent, an OutRecord expires.

PITE is associated with an expiry timer. The expiry timer is fired when the PITE expires. When PITE lifetime is short, it becomes difficult to detect the Interest loop problem, caused by congestion or multi-path propagation. Therefore, another data structure called DeadNonceList is considered for loop detection that complements PIT. The entry deleted from PIT is stored in the DeadNoncelist for some time to address the loopback problem. The entry in the DeadNonceList comprises Interest name and Nonce. Each entry in the DeadNonceList is associated with the static 6 s time value. When an Interest is received, its Nonce and the name is checked in the DeadNonceList. If the matching entry is found, the loop is suspected and the Interest is considered invalid. If a matching Nonce is not found in the DeadNonceList, it searches PIT for the existing entry. If the PITE already exists, the incoming Interest Nonce is checked in the existing PITE before it is processed further. Initially, for each new PITE, the PEL is set by considering the InterestLifeTime field in the Interest. InterestLifetime is specified in the Interest packet header by the consumer before forwarding the packet. The PITE is removed when the PEL expires or the vehicle with PITE receives the required Data packet. In Vanilla NDN, the PEL default value is 4 s.

When a new interest is received and its name matches the PITE, then its Nonce is compared to the Nonces in PITE InRecords. If the Nonce in the new Interest matches the Nonce in an InRecord with similar face, the Interest is considered a legitimate retransmission because there is no risk of a persistent loop. The Interest message is considered as a duplicate in case the Nonce matches the Nonce in an in-record of a different face. If a similar Nonce is not found, the Interest is considered as a new valid Interest. When a new valid Interest for the same name arrives, the expiry timer on the existing PITE is extended. Upon receiving Interest, if no matching PITE is found, then the cache (content store) is checked for the required content. If the required content is not in its cache, an InRecord is added with the incoming face to the PIT. Afterwards, the Interest message is forwarded and OutRecord (which represents the upstream face for the Interest) is added to the PITE. Otherwise, if the required content is found in the cache, the Data message is forwarded with the relevant content to the interfaces saved in InRecord and finally PITE is removed. In native NDN, after receiving a Data message it will be processed only if one or more relevant entries are present in the PIT. If no relevant PITE is found, the received Data message will be considered unsolicited. Unsolicited messages are simply dropped in native NDN. If a relevant PITE is found, the content is stored in the cache before forwarding the Data message. Content admission, lifetime, and replacement are governed by the cache admission and replacement policy.

The consumer vehicle after transmitting the Interest packet waits for a specified period of time for the Data packet. After a specific time period (Retransmission Time Out (RTO)), the consumer retransmits the Interest packet. Consumer vehicles are allowed to make retry attempts before discarding the Interest. Furthermore, in NDN, it is considered that Data packets follow the reverse forwarding paths of Interest packets. In a highly dynamic environment, if the Interest packet kept on moving forward due to the reason that it is not hitting the content provider/producer, then it may be difficult for the Data to be traversed on the same reverse-path as the PEL might have expired on the intermediate vehicles. In native NDN, receiving a Data packet after its PEL expiry is deemed unsolicited. For PIT, a search string prefix-matching algorithm is used, which takes longer due to the large and variable size of the content name. The large size of the PIT will exacerbate the search delay, making it unsuitable for the dissemination of safety content.

## 4. Current State-of-the-Art Schemes

The state-of-the-art PIT management schemes can be broadly divided into two categories: (i) static management, (ii) adaptive management. Table 1 compares existing PIT management schemes. There exists very limited work addressing adaptive PIT management requirements in NDN-based VANETs.

### 4.1. Static PIT Management

There exists work [14,15,16,17,18,19] which considers static PEL. In [14,15,16,17,18,19], each forwarder vehicle stores the PITE for the fixed duration of time. If the Data message is received after PITE expiry, it will be considered as unsolicited.

Source-based communication schemes [14,15] involve communication overhead as they require vehicles to be aware of the topology and physical characteristics of nearby vehicles. Periodic Beacon messages are used to disseminate information. Each vehicle maintains tables and stores relevant information after receiving it from neighbours (such as speed, direction, and connectivity duration). The vehicle then uses the information in these tables to choose the best forwarder. In this way, the broadcast storm problem is addressed in source-based schemes.

Moreover, receiver-based forwarding schemes are also presented in the literature to avoid congestion in the not-safety applications [16,17,18]. Such schemes consider static PEL. The receiver-based forwarding scheme considers assigning priorities to the vehicles in the neighbourhood. A small waiting time is assigned to high-priority vehicles. The vehicle’s suitability to be selected as a forwarder is determined based on its probability in the least waiting time. The vehicle with the lowest deferred timer value is able to forward the Interest message first. All other neighbouring vehicles, upon overhearing the message with the same Nonce and Name (not present in the DeadNonceList), already scheduled for broadcast, stop their timer and back-off from broadcasting duplicate messages. This occurs, for example, in schemes where the forwarder is selected based on the distance to previous forwarder [16,17,18]. Compared to neighbouring vehicles, a lower deferred timer value is calculated for vehicles far from the previous forwarder vehicle. This deferred timer-based rebroadcast mechanism reduces message collision chances and lowers bandwidth usage. Ullah et al. [19] proposed a scheme for caching content near the consumer to reduce the content retrieval delay. This aims to reduce the overall number of PIT entries. The higher the ISR, the less time PIT will keep entries for. The scheme introduced three new fields in the Interest and Data packets: chunk-threshold, hop-count, and TTL. The content holder node inserts the number of pieces available with the content requested by the requester into the chunk threshold. The requester uses ChunkTHd as the priority for conducting Interest prefetching.

In a VANET dynamic environment, a high number of Interests and a large PEL can exhaust the PIT, impacting the overall ISR. In the case of using constant PEL, the PIT must keep the PITE for its associated PEL duration. VANETs applications have varying latency requirements; exploiting similar PEL will not be feasible. With a static PEL, the size of the PIT may grow rapidly because all Interests may not be satisfied due to the unstable wireless environment. Moreover, considering parameters such as speed and direction, the vehicle with PITEs might not receive the Data message, resulting in an increase in the load on PIT. In V2V scenarios where the request is met by other vehicles, when the vehicle still holds the PITE, its performance will degrade due to the large PIT table. The large size of the PIT will exacerbate the search delay, making it unsuitable for the dissemination of safety content. This problem becomes worse in the case of PITE long lifetimes, which increases the number of stale entries in PIT. Stale PIT entries not only waste PIT storage but also increase search time. PIT is a key player in finding content in NDN. Due to the limited opportunities offered by current memory technologies, PIT size is a bottleneck. PIT overflow results in service disruptions as new Interest messages cannot be added to PIT. It is also possible that a consumer vehicle changes its direction or speed and no longer remains within communication range of the intermediate forwarder vehicle, or that it moves away from the path traversed by the interest packet. Such events are very common in VANETs due to high mobility, especially in urban mobility scenarios. Moreover, a shorter PEL will also cause frequent re-requests by consumers, resulting in network congestion and increased ISD.

### 4.2. Adaptive PIT Management

There exists very limited work related to adaptive PIT management in NDN-based VANETs [20,21,22]. Bouk et al. [20] proposed an adaptive PEL scheme. Three information components were used: PEL, rate of decay, and ISR. Initially, the default PEL value was used. PEL depends on two constant information components that are input by consumers: PEL and rate of decay. In this work, no technical details were provided related to the computation of these constants. PEL is reduced at each forwarder vehicle in the upstream direction using the exponential decay model. The proposed scheme is impractical because it considers the fixed (static) initial PEL and the rate of decay.

Manisha et al. [21] proposed a scheme to compute the PEL adaptively at every vehicle, based on the total time duration required to transmit a packet to neighbours in one hop. Three different types of delays are considered: transmission delay, contention delay, and propagation delay. Different VANETs applications have different latency requirements. Additionally, VANETs characteristics make it very difficult to predict Round-Trip Time (RTT). It is believed that the RTT correctly predicts congestion in the end-to-end network path. When considering a wireless network, this belief may be incorrect. Because of channel fading, interference, and mobility, the quality of the radio link in wireless networks can vary greatly over time.

Bouk et al. in another paper [22] used hop count in the Interest message to limit the number of hops an Interest message can traverse. A Hop-limit-based adaptive PEL (LAPEL) scheme is presented for NDN-based VANETs. It computes a one-hop PEL based on the following parameters: contention window, (ii) back-off period, (iii) transmission delay, (iv) propagation delay. The concept of TTL is exploited for the following purposes: (i) to limit the Interest broadcast scope, and (ii) to estimate the PEL at each node. The decay rate between the consumer node and the last Interest-receiving node is adaptively computed using a logistic model. In a dynamic environment, it is very difficult to accurately estimate the time of the round trip.

The schemes presented in [21,22] do not consider forwarding (queuing) delay and processing delay in the model. Receiver-based schemes introduce delays (wait period) to address the broadcast storm problem (which results in congestion and contention). As the packets are delayed for an unknown amount of time, it is hard to predict position, hop count, and RTT. For example, in Duarte et al. [16], the Interest-forwarding vehicle is considered as the one which is farthest from the previous forwarder compared to neighbouring vehicles. The deferred time is calculated based on relative vehicle distance to the content provider/producer and network density. If the vehicle does not overhear the packet during the wait period, it forwards the packet upon timer expiry. In [23], the authors discussed that adaptive PEL schemes must consider the processing delays while computing the PEL. Not considering processing delays might result in the degradation of ISR in some applications. For example, non-trivial computing in NDN-based edge computing will affect the PITE expiry time, leading to the degradation of ISR. Furthermore, in source-based forwarding schemes [14,15], the vehicle collects information from the neighbouring vehicles for forwarder selection. Afterwards, rankings of itself and neighbouring vehicles are computed to select the forwarder. This ultimately adds to processing delays.

Alubady et al. [24,25] proposed an adaptive PIT management solution for the emergency environment. The proposed scheme used a Smart Interest Lifetime (SIL) for PIT overflow management based on the network load. For network load calculation, an extra field termed face list is maintained in the PIT, which holds the number of Interests per interface. Two information components are exploited for PEL threshold computation: (i) the maximum number of Interests received on a particular interface, and (ii) the total number of entries in the PIT. In the event of PIT overload and similar Interest rates on all faces, the lifetime threshold is computed based on the average of PITEs lifetime in PIT. In the case of PIT overflow and dissimilar Interest rates on all the faces, the face with the maximum number of received Interests is considered. The lifetime threshold is calculated by taking the average of all PITE lifetimes (received on face f). In the case of network overload, a shorter PEL value, i.e., a minimum of lifetime threshold and PEL, is considered. In [24,25], application type and scope are not considered while updating PEL.

Alubady et al. [26] proposed the Highest Lifetime Least Request (HLLR) protocol to optimize the PIT performance in case of high network traffic. In [27], the author proposed a PITE replacement policy in CCN networks considering a natural disaster application scenario. In [10], the PIT entry replacement scheme is presented, which is based on the Highest Lifetime least Request Policy (HLLR) presented in [26,27]. Upon receiving the Interest, if the PIT is full, the PITE with the longest remaining LT and the fewest requests is replaced with a new one. In the event of PIT overflow, the scheme replaces the PITE for the upcoming Interest with the PITE with a maximum lifespan and a minimum number of incoming faces. The replacement decision is based on the following components: (i) number of incoming interfaces, and (ii) Interest lifetime. The PIT replacement policy does not consider the time PITE spent in the PIT as a factor in determining the lowest priority PITE (PITE in the PIT) to be replaced. It is possible that the PITE in the PIT which is selected for removal might be close to satisfaction. The scheme may replace the PITE in the PIT, which might be satisfied in the following few milliseconds. Not considering the time spent on each PITE can affect PIT utilization. In [10], the PITE adaptive lifetime policy is also presented, where the PEL is managed as follows. When the PIT is empty, the PEL threshold is set as an average of the value specified in the incoming Interest packet InterestLifetime field and the default lifetime value. In case the PIT is not overflowed and not empty, the PEL threshold is updated by calculating the average PEL between the PEL threshold value and the value specified in the incoming Interest packet InterestLifetime field.

PIT is an important player in NDN data retrieval. PIT size is the bottleneck due to the limited opportunities provided by current memory technologies. PEL adjustment is a critical challenge that can have an impact on overall network performance. There are adaptive PIT management schemes that use the following parameters to reduce the lifetime of the PITE between the consumer node and the last Interest receiving node: hop count [20,22], Round-Trip Times (RTT) [20,21,22], and Interest Satisfaction Rate (ISR) [20]. In VANETs, the hop count, network density, and RTT [20,21,22] are unpredictable. Whereas ISR also depends on the underlying caching mechanism’s efficiency. Due to the transient communication nature, the time of vehicles’ interconnection cannot be predicted. Speed variation makes estimation of propagation delay difficult. To address the broadcast storm problem, receiver-based forwarding schemes introduce delays (waiting time) at each hop. As discussed above, receiver-based schemes introduced delays (wait period) to address congestion. As the packets are delayed at different hops for an unknown amount of time, it is hard to predict forwarding (queuing) delay and RTT. The presented schemes [21,22] do not consider queuing delay and processing delay in the model. Vehicles are mobile and pass through areas with different densities, topography features, and wireless conditions. This makes the prediction of contention delay difficult. Not considering processing delays might result in the degradation of ISR in some applications. Likewise, not considering the content type [10,26,27] while replacing PITE could lead to life loss and disabilities.

**Table 1:** PIT Management Schemes in NDN-based VANETs.

Ref.	PIT Management	PIT Management Strategy	Simulation Environment	Limitations
[14,15]	Static	□ Neighbour-based forwarding selection strategy □ PIT entries are managed by selecting limited forwarder(s)	□ NS-2, □ Highway scenario with mobility	□ Contention problem due to periodic Beacon overhead □ Stale PIT entries in high density networks.
[16,17,18]	Static	□ Receiver-based (position-based) forwarding. □ PIT entries are managed by forwarder selection	□ ndnSIM, □ Urban scenario with mobility	□ Stale PIT entries due to more chances of Reverse Path Partioning problem as distant neighbor is selected as forwarder.
[19]	Static	□ To proactively cache the content near to the requester to reduce the number of PIT entries	□ ndnSIM, with infrastructure environment	□ Communication overhead due to extra fields in Interest/Data packets □ Static *PITE* lifetime □ Wastage of storage due to replication of content
[10]	Adaptive	□ *PITE* Replacement policy is same as presented in [26,27]. □ Adaptive PEL scheme proposed (i) When PIT not overflow - Threshold is set as an average of Interest LT and default LT. (ii) When PIT is in overflow □ PEL threshold is updated by calculating the average LT between the LT threshold value and the incoming packet LT.	□ ndnSIM □ No mobility □ Rocket fuel mapped topology	□ Application type, content type, application popularity not considered. □ The received valid Interest *PITE* is replaced with the *PITE* in the PIT which might be about to be satisfied. □ Type of content, and environmental conditions not considered.
[20]	Adaptive	□ Adaptive PEL scheme based on three information components: PEL, decay rate, and ISR.	□ NS-2, Highway scenario with mobility	□ PEL depends on two constant information components that are input by consumer: PEL and rate of decay. No technical details are provided related to computation of these constants. □ Proposed scheme is impractical as it considers fixed initial PEL and the decay rate.
[21]	Adaptive	□ Adaptive PEL based on the total time duration required to transmit a packet to neighbours in 1 hop. Three types of delays are considered: Transmission delay, contention delay, and propagation delay.	□ ndnSIM, Highway scenario with mobility	□ Not considered processing and queuing delays □ In VANETs, it is very challenging to predict round trip delay
[22]	Adaptive	□ Hop-limit-based adaptive PEL scheme is presented for NDN-based VANETs. □ It computes 1-hop PEL based on the following parameters: (i) contention window, (ii) backoff period, (iii) transmission delay, (iv) propagation delay.	□ NS-2, □ Highway scenario with mobility	□ Based on hop count rather than type of content. □ Specific application scenario is considered. □ Queuing and processing delays are not considered while computing delay to adjust PEL.
[24,25]	Adaptive	□ PEL updating of incoming Interest in the event of PIT overflow □ In case of similar Interest rate on all faces, an average lifetime of all the PITEs in PIT is calculated to represent a new lifetime threshold □ In case of dissimilar Interest rate on all the faces, the average lifetime of all PITEs for face f is calculated to represent lifetime threshold. Face f represents the face with maximum number of received Interests. □ PEL of incoming Interest is set as minimum of PEL and lifetime threshold.	□ Not Discussed	□ Content type, spatial and temporal validity, and environmental conditions not considered while computing PEL of incoming Interest. □ A dedicated scenario is considered. □ Reducing the PEL for critical content might lead to loss. □ Very short Interest lifetime of 80 ms [25]
[26,27]	Adaptive	□ The *PITE* replacement policy replaces the *PITE* for the incoming Interest with the *PITE* in PIT with the lowest priority (maximum lifespan and a minimum number of incoming faces).	□ ndnSIM, grid-based scenario with no mobility	□ Dedicated scheme considering disaster not for general/infrastructure-less environment □ The received valid Interest *PITE* is replaced with the *PITE* in the PIT which might be about to be satisfied. This in turn impacts PIT utilization. □ Type of content and application popularity not considered.

## 5. Context-Aware Pending Information Table Entry Management (CPITEM) Scheme

Packet drop and delay in VANETs is common and can lead to a maximum number of stale PIT entries. Chances of Data packet loss are higher because of their size. The most important challenge in PIT management is to decide which PITE to be replaced, or life decreased in case of PIT overload. Removing a PITE might disrupt some critical services. Demand for safety and non-safety information in VANETs is increasing significantly. Drivers and passengers have a higher demand for traffic-related information, popular content as well as road entertainment applications, such as weather information, breaking news, streaming live videos, and downloading multimedia content. Different delivery priorities may be required. For example, video delivery to a vehicle required for safe driving (to raise awareness related to an area of interest under bad weather) should take precedence over the delivery of a recent cartoon for a child. The later video is less critical. This in turn requires that the PITE for safety content be given priority over the PITE for non-safety content. In the event of PITSize>threshold(θ), PITE related to a safety application must not be replaced.

PIT management is critical for effective content distribution. To meet the latency requirements of VANET applications and effective management of PIT, PITEs must be prioritised. Furthermore, allocating priorities will aid in decision-making regarding PITE replacement in the event of PIT overload. Considering VANET applications’ QoS requirements, assigning priority to PITE is critical. However, the question is which components of information should be considered to prioritize PITEs.

The CPITEM scheme, therefore, provides the mechanisms for the context-aware PITE replacement in case of PITSize>threshold(θ). In the VANETs, different applications have different QoS needs. In the proposed CPITEM scheme, the PITE priority is calculated using the following information components: (i) Content type (safety/non-safety), (ii) Application popularity (demand of application), (iii) PITE lifetime (PEL), (iv) PITE size in terms of InRecords associated with PITE, and (v) duration of time PITE stays in PIT (termed resting time in PIT). When a vehicle receives an Interest packet, it first checks the PIT. If it is valid message and there is a space in the PIT store, the PITE will be stored directly. Otherwise, the CPITEM scheme computes the priority based on aforementioned components. If the received Interest priority is greater than the PITE in the PIT with lowest priority, the PITE in the PIT with lowest priority will be replaced with the PITE for the received Interest. Otherwise, if the upcoming Interest has a lower priority than the PITE in the PIT with the lowest priority, the newly received Interest will be dropped.

Likewise, the original NDN forwarding daemon, the consumer vehicle, sends an Interest message containing the default attributes including Nonce, InterestLifetime, and hop count. Only the Data name is constructed based on our previously proposed context-aware content-naming scheme [9]. The CACN Data-naming scheme allows for the identification of both safety and non-safety contents. Furthermore, the CACN features a coding scheme that represents the majority of the content name components, allowing for addressing communication and storage complexity [9]. The CACN scheme allows for representing Content Type (CT), Content Scope (CS), and Application ID (AppID) information in the content name along with other information components. The CACN is divided into two partitions: (i) obligatory and (ii) supplementary. The information about the content is stored in the obligatory part. The context information considers the following information components: content type, content scope, content format, application, when, and where. Content type represents the kind of content, i.e., safety or non-safety. Content scope represents the scope of the content, i.e., local or global. The content field represents content format, which is divided into four categories: text, audio, image, and video. The application component uniquely identifies the VANETs application.

### 5.1. Content Type

Non-safety applications aim to improve the comfort levels of drivers and passengers and make travel more pleasant. Compared to safety applications, most of the non-safety applications do not have stringent low-latency requirements. On the contrary, the quality of service (QoS) required by the safety apps is close to real-time. Delaying the dissemination of information in some safety applications can lead to loss of life and disability. These applications and services are bound to low-latency needs. Therefore, considering the type of content plays an important role in prioritizing PITE. Most of the safety applications do have local CS as they have limited spatial validity. In addition, these applications have limited time validity, after which the content is not considered valuable. Considering time validity, safety content PEL would be short compared to non-safety content. Therefore, safety content will create less load on PIT compared to non-safety content. The content type attribute in the CACN can be exploited to compute the content type of content requested in the Interest message. If in the Interest name the CT is specified as safety, then that PITE should be given priority over the non-safety content PITE. Additionally, unlike [10,24,25], its lifetime also should not be reduced. In case of PIT overload, if the received valid Interest is related to safety content, its PITE must be replaced with existing PITE of non-popular, low-rated, non-safety content.

### 5.2. Non-Safety PITE Priority

In our proposed CPITEM scheme, in the event of PIT overload, if the received Interest i PITE ($PITE_{i}$) does not already exist in the PIT and the application type is non-safety, the $PITE_{i}$ will be replaced with existing non-safety $PITE_{j}$ having lowest priority. In case all the entries in the PIT have higher priority compared to the priority of incoming Interest, the $PITE_{i}$ will be dropped. The PITE priority is computed considering the following information components: (i) Eminence of the $PITE_{i}$ ($Eminence_{PITE_{i}})$, (ii) $PITE_{i}$ Utility ($Utility_{PITE_{i}}$), (iii) Application priority ($Utility\text{\_}App_{i}^{t_{r}}$).

Upon receiving an Interest packet, PIT is searched for in the existing entry. If no PITE is found, entry is created along with the incoming interface (InFace). If the PITE exists and has an InRecord with a similar content name and Nonce, then the Interest packet is simply discarded. On the other hand, if the PITE exists and has an InRecord with a different Nonce but with the same content name, then the aggregation of InFace and Nonce takes place in the PITE. When a new valid Interest for the same name arrives, InRecord is added to the PITE.

#### 5.2.1. Eminence

$Eminence_{PITE_{i}}$ is computed using Equation (1) considering the information components: (i) remaining lifetime of $PITE_{i}$ ($PEL_{PITE_{i}})$, (ii) size of the $PITE_{i\;}(Size_{PITE_{i}})$, (iii) remaining lifetime and size of all entries in the PIT. $Size_{PITE_{i}}$ is the number of InRecords associated with a $PITE_{i}$, representing the current demand for the content. Eminence represents the popularity of PITE by considering both PITE size and PEL. To compute the popularity of the current PITE, we have to divide it by the summation of the product of the size and PEL of all PIT entries ($\overset{k}{\underset{j=1}{\sum }}(Size_{PITE_{j\;}}*PEL_{PITE_{j}}).$ If two PITEs have equal PEL ($PEL_{PITE_{j}}==PEL_{PITE_{i}})$, lower Eminence value is calculated for the PITE with smaller size (less number of InRecords), whereas higher Eminence value is calculated for the PITE with larger size. Some of the PITEs may have a large size and a large PEL, resulting in a higher Eminence value, which creates a load on the PIT. Therefore, PITE utility (Equation (2)) is computed to prioritise those PITEs that have a large size but have spent more time in PIT. If two PIT entries $PITE_{i}$, and $PITE_{j}$ have nearly equal Eminence values, then a higher Utility value will be computed for PITE which rests in PIT for a longer duration. This is due to the fact that the entries that spend a longer time in PIT might be resolved sooner. Consequently, the PIT storage will be available for the upcoming PIT entries. This in turn will improve PIT utilization. The Eminence of the PITE is computed as follows.

(1) EminencePITEi=(SizePITEi ∗PELPITEi)∑j=1k(SizePITEj ∗PELPITEj)

#### 5.2.2. Utility

The $PITE_{i}$ Utility is computed using Equation (2), considering $Eminence_{PITE_{i}}$, rested time in PIT by the $PITE_{i}$ ($RT_{PITE_{i}}$), and $PEL_{PITE_{i}}$. $Utility_{PITE_{i}}$ takes into account PIT utilization. The $PITE_{i}$ which has spent more time in PIT is given preference. This in turn will not only help to reduce average Interest Satisfaction Delay (ISD) but also PIT utilization. The $PITE_{i}$ $with\;a\;large\;Size_{PITE_{i}}$ and $RT_{PITE_{i}},$ will be given high priority. If two PIT entries $PITE_{i}$, and $PITE_{j}$ have same size ($Size_{PITE_{i}}==Size_{PITE_{j}})$, then PITE which rested in PIT for a longer duration will be given a higher utility.

(2) UtilityPITEi=EminencePITEi(PELPITEi/RTPITEi)

#### 5.2.3. Application Popularity

The application attribute in the CACN can be exploited to compute the application ID of content requested in the Interest message. The popularity of the non-safety application $App_{i}$ indicates whether the content requested by the consumer vehicle is in demand in the geographic region. If the application $App_{i}$ is popular, it indicates that the PITE associated with it is more valuable and relevant. Likewise, there is a greater likelihood of obtaining the content in the vicinity. Application popularity may be calculated based on the number of Interests received during a certain time period on the face(s). CACN with its coding scheme can represent a wide range of safety and non-safety applications. For this purpose, the AppID information component is exploited. Since vehicles in the VANETs are mobile, passing through different geographical areas, different applications contents can be in demand. Moreover, an application may be popular for a certain time and then may be disregarded in a spatial region. If the scheme assigns equal weightage to the Interest messages received over time, requests for popular content may not be addressed. It would be difficult to differentiate between the situation whether the application is high in demand currently or was in demand previously. Let us consider an example scenario, the application $App_{i}$ is highly demanded (a large number of Interest messages received) at a certain time period while moving through some geographical area. After the passage of some time $t_{2}$, let us consider that a large number of requests are received for application $App_{j}$, but no additional Interests are received for $App_{i}$. If the count of Interests received for $App_{i}$ is greater than $App_{j},$ it will still be considered as a highly demanded application. As a result, when calculating application priority, the time-sensitivity requirement must be taken into account. If the time-sensitivity requirement is not taken into account, it would be difficult to differentiate between whether the application is high in demand currently or was in demand previously.

The time sliding window [28,29] is used to address the time-sensitivity requirement when calculating application popularity. Let us consider that for an application $App_{i}$ recent time window $App_{i}\text{\_}t_{r}$, the dimension is in the form [FROM Start, TO End]. Each record in the time window is kept for the duration t using time unit (SECOND (s), MINUTES (m)). When a vehicle receives an Interest, considering its arrival time, its expiry time is calculated and it will store a tuple in the $App_{i}\text{\_}t_{r}$. At the expiry of the timer associated with each entry in $App_{i}\text{\_}t_{r}$, the entry will be removed from the $App_{i}\text{\_}t_{r}$.

Let us consider that the current PIT entries for non-safety content belongs to n applications. Application priority is represented with Utility of $App_{i}$ at time t ($Utility\text{\_}App_{i}^{t_{r}})$. The $Utility\text{\_}App_{i}^{t_{r}}$ is calculated using Equation (3). $Utility\text{\_}App_{i}^{t}$ represents the current demand of an application $App_{i}$. $Intfreq\text{\_}App_{i}$ represents the number of valid Interests received for $App_{i}$ in time window $App_{i}\text{\_}t_{r}$. $\overset{m=n}{\underset{m=1}{\sum }}Intfreq\text{\_}App_{m}$ represents the count of valid Interest messages received for each of the n applications in the recent time period t.

(3) Utility_Appitr=Intfreq_Appitr∑m=1m=nIntfreq_Appmtr

$Utility\text{\_}App_{i}^{t_{r}}$ denotes the relationship or comparison between the Interest messages received for a specific application and the total number of Interest messages received for all applications. It compares the two quantities with respect to each other. Higher $Utility\text{\_}App_{i}^{t_{r}}$ is calculated for an application ($App_{i}$) for which a larger number of Interest messages are received compared to an application with a lower number of Interest messages.

#### 5.2.4. PITE Priority

By taking both $Utility_{PITE_{i}}$ and the popularity of the application $(Utility\text{\_}App_{i}^{t_{r}}$), the priority of the $PITE_{i}$ is computed using Equation (4). $Priority_{PITE_{i}}$ considers both application utility and PITE utility. If two PIT entries have the same size and application utility, then in the event of overload, the PITE that rested in PIT for a shorter period of time will be chosen for replacement.

(4) $$Priority_{PITE_{i}}=Utility_{PITE_{i}}+Utility\text{\_}App_{i}^{t_{r}}$$

Furthermore, the following algorithms (Algorithms 1–3) related to the CPITEM scheme are presented, which are executed at the reception of Interest messages.
**Algorithm 1:** Context-aware PIT Entry Management (CPITEM) Protocol1  *// Interest message (IntMsg) include content name* (*CACN*)2  *Upon Receiving IntMsg*3   *nonce* ←IntMsg.*Nonce*4   *interestName* ←IntMsg.*Name*5  * Face* ←*IntMsg.InFace*6   *AppID*←*GetAppID(interestName)*7   If (Valid*PITEExistsInPIT*(*interestName*) == *False*)8    //*Associate time t with new entry related to Application*9    *UpdateAppInf*(*AppID,CurrentTime*(),*t*)10      If ((*ContentInCache*(*interestName*) == *False*))11      *If PITSize*(*PIT*) < *threshold*12        *AddPITE*(IntMsg)13        Set IntMsg to *Forward*14       Else
15        *Contextaware_PITE_Replacement*(IntMsg)16      *Else*17        *Construct Data message and forward*18   *Else*19      If (*PITEExistsinPIT(interestName*) == *True*)20      * PITE*←*SearchPITE*(IntMsg)21       If (*ValidInRecord(interestName,Nonce,Face,PITE*) == *True*)22         *AddInRecToPITE*(*PITE*)23         *//Associate time with new entry related to Application and add it to related time window*24         *UpdateAppInf*(*AppID,CurrentTime*(),*t*

When an Interest is received, its Nonce and name are checked in the DeadNonceList. If the matching entry is found, the loop is suspected and the Interest is considered invalid. If a matching Nonce is not found in the DeadNonceList, it searches PIT for the existing entry (Line 7, Algorithm 1). When a valid Interest is received for which no PITE already exists and related content is not present in the cache, the PIT size is checked. If the PIT size is less than a predefined threshold, the PITE is created and stored in the PIT. Otherwise, the Contextaware_PITE_Replacement method (Algorithm 2) is executed. If the required content is found in the cache, the Data message is forwarded with the relevant content, and finally, PITE is removed. For each valid Interest, the corresponding application window is also updated by adding a time-bound application-related tuple (Line 9, Algorithm 1). If the PITE already exists, the incoming interest Nonce and face is checked against the existing PITE before it is processed further. If a similar Nonce is not found, the Interest is considered as a new valid Interest and InRecord will be added to the corresponding PITE (Lines 21–22, Algorithm 1). When a new valid Interest is received, its application ID (AppID) is checked from CACN. Afterwards, after associating the time t, the tuple is added to the relevant application time window. The tuple will be removed from the corresponding time window after the expiry of time.
Algorithm 2: Contextaware_PITE_Replacement (IntMsg)1   // Interest message (IntMsg) include content name (CACN)
2       interestName←IntMsg.Name
3     CT←GetContentType(interestName)
4     If (CT==Safety)
5      PITE, Priority ←LowPriorityNonSafetyPITE()
6      If (PITE !=Null)
7          RemovePITEinPIT(PITE)
8     AddPITEInPIT(IntMsg)
9      Set  IntMsg  to Forward
10     Else11     * // All PITE are related to safety in PIT. No space for new entry*12      Discard(IntMsg)
13     Else14      PITE, Priority ←LowPriorityNonSafetyPITE()
15      // ComputePriority(IntMsg) computes recent  IntMsg PITE Priority using Equation (4)16      Priority1 ← Compute Priority(IntMsg)
17      If (Priority1>Priority)
18       RemovePITEinPIT (PITE)
19       AddPITEInPIT(IntMsg)
20       Set  IntMsg  to Forward   
21       Else22       *// All PITEs in PIT have high priority compared to received message PITE priority. No space for new entry*23       Discard(IntMsg)


If the content type is safety, low priority non-safety PITE is searched in the PIT (Line 5, Algorithm 2). In our proposed CPITEM scheme, in the event of PIT overload (PIT size is greater than a certain threshold), if the received valid safety Interest PITE does not already exist in the PIT, the PITE will be replaced with the existing non-safety $PITE_{i}$ in the PIT having the lowest priority. If all of the entries in the PIT are related to safety applications, the incoming Interest message (IntMsg) will be simply dropped in the event of PIT overload (Line 12, Algorithm 2). If the received Interest message is of type non-safety (Line 13, Algorithm 1) the priority of the incoming Interest PITE and priority of the lowest non-safety PITE in the PIT will be compared. If the priority of the incoming Interest PITE is greater than the priority returned by the method LowPriorityNonSafetyPITE(), then the corresponding PITE in the PIT will be removed from the PIT. Afterwards, PITE for the incoming Interest will be added (Line 19, Algorithm 2). ComputePriority(IntMsg) method computes the priority of incoming Interest PITE considering Equation (4). Algorithm 3 illustrates the working of the LowPriorityNonSafetyPITE() method. If one or more non-safety PITEs exist in the PIT, this method returns the lowest-priority non-safety PITE reference and its priority. This method computes the priority of every PITE in the PIT considering Equation (1) to Equation (4). Afterwards, the non-safety-content PITE with the lowest priority is searched and returned.
**Algorithm 3:** LowPriorityNonSafetyPITE()1    // Interest message (IntMsg) include content name (CACN)
2   
PITLoad ← 0
3    interestName←IntMsg.Name
4    $\;\;\;\;\;For\;each\;PITE_{j}\;\in PIT$
5      PITLoad ← PITLoad + $\left(Size_{PITE_{j\;}}*PEL_{PITE_{j}}\right)$
6   LowestPITEPriority ← β //  β is a Highest Value
7    LowestPITE ← Null
8    $For\;each\;PITE_{i}\;\in PIT$
9       AppID ←GetAppID(interestName)
10     $Eminence_{PITE_{i}}\;$←(SizePITEi ∗PELPITEi)PITLoad
11     $Utility_{PITE_{i}}\;$←EminencePITEi( PELPITEi/RTPITEi)
12     // Compute Application Popularity using Equation (3)13     Utility_App ← ComputeAppUtility(AppID)
14       $Priority_{PITE_{i}}\;\leftarrow \;Utility_{PITE_{i}}+Utility\text{\_}App$
15       If ($Priority_{PITE_{i}}<$ LowestPITEPriority)
16       
$LowestPITEPriority\;\leftarrow Priority_{PITE_{i}}$
17       
$LowestPITE\;\leftarrow \;PITE\left(PITE_{i}\right)$
18   
Return LowestPITE, LowestPITEPriority


## 6. Performance Evaluation

For evaluation of our proposed scheme called the CPITEM protocol, which exploits the CACN naming scheme, we implemented it in ndnSIM [11]. We compare our scheme’s CPITEM performances with the HLLR [10,26,27]. A similar PIT-replacement scheme is presented in [10,26,27]. In the event of a PIT overflow in HLLR, the PITE with the longest lifetime and fewest requests is replaced with a PITE of the newly received Interest.

In case of CPITEM, likewise, the original NDN-forwarding daemon, the consumer vehicle, sends an Interest message containing the default attributes including Nonce, InterestLifetime, and hop count. The Data name is constructed based on our previously proposed context-aware content-naming scheme [9]. The CACN Data-naming scheme allows for the identification of both safety and non-safety contents. Furthermore, the CACN features a coding scheme that represents the majority of the content name components, allowing for addressing communication and storage complexity [9]. To investigate the impact of shared communication channels, a highway traffic scenario is used to simulate the performance of the HLLR [11,12,13] and proposed CPITEM scheme. The highway scenario consists of a 10 km, four-lane road. In this scenario, the consumer vehicles transmit the Interest message to the vehicles in its neighbourhood. Two safety and four non-safety applications are considered. Consumers are requesting content by sending Interest messages, whereas the producers who have the required content send the relevant Data messages after receiving the Interest messages. Consumers and producers are both mobile. Six applications types are taken into account: post-crash application, work-zone application, file sharing application, commercial advertisement application, parking availability application, and traffic navigation map application.

Both schemes consider a pull-based scheme for obtaining the required content from the content holder. Just like in the work of [9], we assume that each vehicle is equipped with GPS, via which it obtains its location. Common parameter settings for all the experiments are depicted in Table 2. Producers, consumers, and forwarders are all mobile. Speed is assigned randomly to vehicles. Due to the variation in speed, a dynamic environment is generated that limits inter-connectivity between vehicles for a longer period of time.

There are seven producers, and each produces content related to a specific application. All the contents related to one application are stored on one producer. The producers are located at different positions on the highway between 1.1 km and 2.4 km. Safety content producers are located between 1.1 km and 1.35 km. Considering the time and spatial validity requirements of the applications, the producers of the safety content are placed closer to consumers (1.1 km) compared to the non-safety content (located at a distance between 1.3 km and 2.4 km). Therefore, the PIT entries for safety content are resolved a little earlier compared to non-safety content.

Two safety application contents and five non-safety application contents are considered. In Safety Content Application (SCA) scenarios, the consumers are sending Interest messages to request safety content related to applications SCA1 (Post crash) and SCA2 (work zone). Considering our proposed CACN scheme, two different safety content names are exploited for safety applications, which include: (i) SCAI (Safety/Local/Text/Post−crash:Head−on Collisions/….), (ii) SCAII (Safety/Local/Text/ Road congestion:Work Zone/….). In Non-Safety Content Application (NSCA) scenarios, the consumers are sending Interest messages to request non-safety content related to applications such as NSCAIA (multimedia file sharing), NSCAIB (multimedia file sharing), NSCAII (commercial advertisement), NSCAIII (navigation map), and NSCAIII (parking availability). Considering our proposed CACN scheme, the five different non-safety content names considered for experimental evaluation are as follows: (i) NSCAIA (Non−Safety/Global/Audio/Multimedia file sharing:Music/….), (ii) NSCAIB (Non−Safety /Global/Video/Multimedia file sharing:Drama/….), (iii) NSCAII (Non−Safety/Local/Text/Commercial Advertisement:Hotel/….), (iv) NSCAIII (Non−Safety/Local/Pictorial/Navigation Map:City/….), and (v) NSCAIV (Non−Safety/Local/text/Parking availability:/….). There are eight consumers who are requesting content at the same rate. In the case of the multimedia file sharing application, three consumers are sending Interests for two types of content (NSCAIA, NSCAIB). Two consumers are showing interest in NSCAIA content, and one consumer is interested in NSCAIB content. The multimedia file sharing application is the most popular application in the simulation scenario. The reason is that two different multimedia content types are demanded by a group of three different consumers. One of the other five consumers is interested in SCAI content; the second is interested in SCAII; the third in NSCAII; the fourth in NSCAIII; and the fifth in NSCAIV content. After every time $t^{\prime },$ the consumer vehicle sends the Interest packet for the different content related to similar applications. Like HLLR, the NDN default forwarding (Interest broadcast) scheme is used as a forwarding strategy in our experimental demonstration.

### 6.1. Experiment 1: Total Number of Drop Interest Messages Due to PIT Size as a Function of Growth in Interest Messages

This experiment is carried out to demonstrate the total number of dropped messages due to PIT overflow as a function of growth in Interest messages. The network comprises 130 vehicles, 8 of which are consumers, and 7 are producers. The PIT size, which represents the amount of available space in each vehicle in terms of PIT entries, is set at 30 entries. In Experiment 1, for the first point on the x-axis, the message generation rate is four messages/s. For the second, third, fourth, and fifth points, the message generation rate is five, six, seven, and eight messages per second, respectively. The number of messages transmitted varies from 1600 to 3200. When the number of messages transmitted is 1600, 400 messages are related to safety, 600 messages are related to popular non-safety, and 600 messages are related to non-popular non-safety content.

**Figure 1:**
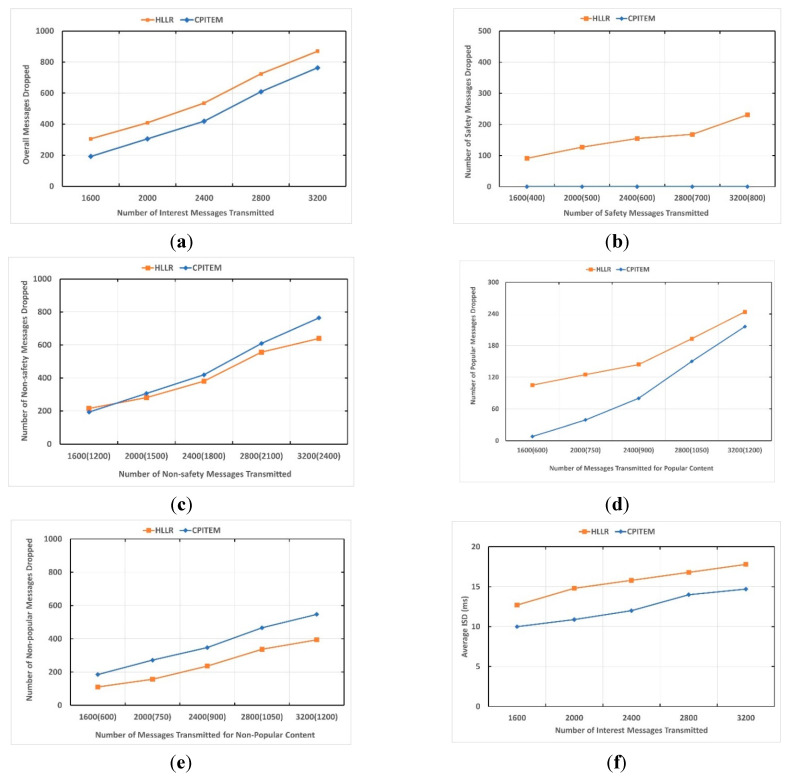
Experiment 1: total number of drop Interest messages as a function of growth in interest messages. (**a**) Overall Interest messages dropped as a function of Interest message growth in the network, (**b**) Interest messages related to safety dropped as a function of Interest message growth in the network, (**c**) interest messages related to non-safety dropped as a function of Interest message growth in the network, (**d**) Interest messages related to popular, non-safety content dropped as the number of Interest messages in the network increases (**e**) Interest messages related to non-popular, non-safety content dropped as a function of Interest message growth in the network, (**f**) average interest satisfaction delay.

Upon receiving the Interest, each vehicle creates a PITE if it is a valid Interest, and the PITE does not already exist. Furthermore, only vehicles with matching PIT entries forward the Data message. Unsolicited messages are simply dropped. Figure 1 depicts how the implemented PITE replacement schemes (HLLR [11], CPITEM) behave as the number of Interest messages in the network increases. Figure 1a depicts the overall number of dropped Interest messages as the number of Interest messages in the network increases. The number of dropped Interest messages for safety content is depicted in Figure 1b as a function of Interest message growth in the network. Figure 1c depicts the number of dropped non-safety Interest messages as a function of Interest message growth in the network. Figure 1d depicts the number of dropped non-safety, popular Interest messages in the network as a function of Interest message growth. The number of dropped Interest messages for non-safety, non-popular content is depicted in Figure 1e as a function of Interest message growth in the network. Figure 1f illustrates the average interest satisfaction delay of HLLR and CPITEM schemes in a VANET highway scenario as a function of Interest message growth in the network.

In this experimental scenario, the producer and consumer vehicles and all other vehicles are mobile. Therefore, the PIT entries are satisfied in a short period of time. To create a load on the PIT, the PIT size is considered small (30 entries). The proposed CPITEM scheme is able to prioritise the PIT entries based on application type (safety, non-safety), time rested in PIT, size (in terms of InRecords), and application popularity. In Figure 1, it can be seen that the proposed scheme prioritises the safety content over the non-safety content. Moreover, CPITEM prioritises popular, non-safety content over non-popular, non-safety content. CPITEM results in a lower number of messages being dropped compared to HLLR. The reason is that a CPITEM drops the incoming Interest if its priority is lower than the lowest priority PITE in the PIT.

To make the multimedia application popular, more Interests are transmitted related to the application compared to other safety and non-safety non-popular applications. Three consumers are sending Interests related to multimedia applications. The PIT size is considered small to create a load on the PIT as vehicles are mobile. In the event of a PIT size being above a certain threshold, CPITEM tries its best to replace the newly received safety content Interest message PITE with non-popular content PITE, as shown in Figure 1b. However, in the event that all entries in the PIT are related to popular non-safety content, the lowest priority popular PITE is replaced with a safety content PITE. Moreover, the PIT size is very limited, and when it is full of popular content entries, the upcoming popular content Interest PITE cannot be placed in the PIT. Therefore, it will be dropped. In Figure 1d, it can be seen that for the CPITEM scheme, some of the PIT entries related to popular content are dropped (due to PIT size) when the load on the network in terms of Interest messages increases.

HRRL, upon receiving each new valid Interest message, replaces one of the existing PITE with the new one, in case of PIT overflow. The HLLR scheme replaces the PITE for the upcoming Interest with the PITE in the PIT with a maximum lifespan and a minimum number of incoming faces. It does not take into account the time PITE spend in the PIT, waiting for the Data packet. This impacts PIT utilization. It is possible that the PITE in the PIT which is selected for removal might be closed to satisfaction, as it is waiting for a long time. Moreover, the newly received Interest PITE priority is not considered. It is always replaced with the existing lowest priority entry (with a maximum lifespan and a minimum number of incoming faces) in the PIT. It can be seen that due to these reasons, the HRRL results in more PIT entries being dropped compared to the CPITEM. Figure 1f illustrates the average interest satisfaction delay of HLLR and CPITEM schemes in a VANET highway scenario. It can be seen that CPITEM results in lower average ISD compared to HLLR.

### 6.2. Experiment 2: Effect of PIT Size

The experiment is conducted to demonstrate the behaviour of CPITEM as a function of growth in PIT size. The network comprises 130 vehicles, 8 of which are consumers, and 7 are producers. Consumers are transmitting Interest messages at a rate of six messages per second. The number of messages transmitted is 2400, with 600 related to safety, 900 related to popular, non-safety and 900 related to non-popular, non-safety content. The PIT size, which represents the amount of available space in each vehicle in terms of PIT entries, varies from 20 to 60 entries. Upon receiving the valid Interest, each vehicle creates a PITE if the PITE does not exist and PIT size is above certain threshold. Furthermore, only vehicles with matching PIT entries forward the Data message. Unsolicited messages are simply dropped.

**Figure 2:**
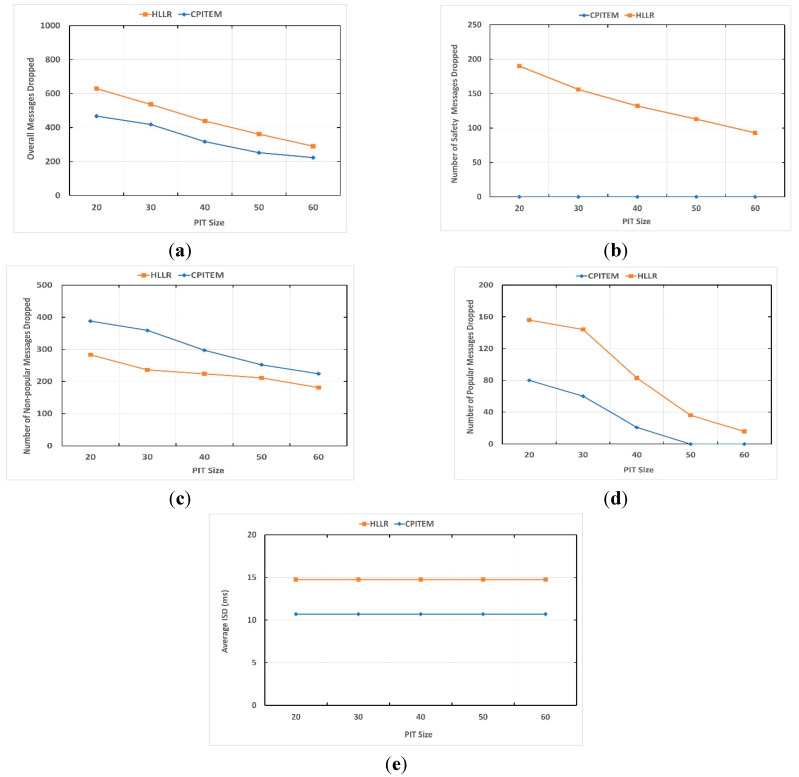
Experiment 2: Effect of PIT size. (**a**) Overall Interest messages dropped due to PIT size, (**b**) Interest messages related to safety dropped due to PIT size, (**c**) Interest messages related to non-popular, non-safety dropped due to PIT size, (**d**) Interest messages related to popular, non-safety content dropped due to PIT size, (**e**) average ISD.

Figure 2 depicts how the CPITEM and HLLR (implemented PITE replacement scheme) behave as the PIT size grows. The number of overall Interest messages dropped as a function of increase in PIT size is depicted in Figure 2a. Figure 2b depicts the number of safety Interests messages dropped as a function of PIT size. Figure 2c depicts the number of non-popular, non-safety contents dropped as a function PIT size variation. Figure 2d depicts the number of popular Interest messages dropped as a function of PIT size variation. Figure 2e depicts ISD as a function of PIT size growth.

Figure 2 demonstrates that considering the load on the network in terms of Interest messages, the CPITEM scheme performs efficiently. Considering the static load and by increasing the PIT size, the number of dropped messages reduces both for CPITEM and HLLR schemes. CPITEM intelligently prioritises the safety content over the non-safety content. No safety related content is dropped by CPITEM. Moreover, CPITEM also prioritises popular content over non-popular content. CPITEM results in reduced average ISD compared to HLLR. HLLR is incapable of distinguishing between safety and non-safety content, as well as popular and non-popular non-safety content.

## 7. Conclusions

The Pending Information Table (PIT) is an important component of the NDN content discovery process. Due to the limited capabilities of today’s memory technologies, PIT size is a bottleneck. Packet loss and delays are typical in VANETs, resulting in a maximum number of stale PITEs. PIT overflow results in service disruptions as new PITEs (of incoming interests) cannot be added to PIT. In VANETs, applications under the safety subgroup include time-sensitive applications. The utility of pertinent information is lost if it is not delivered on time. Safety applications demand near-real-time quality of service. In PIT management, the most important challenge is to decide which PITE to be replaced in the event of PIT overload. Removing the PITE might disrupt some critical services. The current state-of-the-art PIT management schemes remove PIT entries of low priority to make room for incoming Interest messages PITEs. When computing priority, the current state-of-the-art method ignores context such as application type, time rested in PIT, and content type. In this work, we proposed a Context-aware PIT Entry Management (CPITEM) protocol for NDN-based VANETs. It manages the PIT table by taking into account information components such as content type, PITE lifetime, PITE size, time for which PITE rested in the PIT, and application popularity. To demonstrate that the proposed CPITEM scheme is efficient and effective, a set of simulations are run and compared to a benchmark scheme.

## Figures and Tables

**Table 2:** Common Configuration Parameters.

Parameters	Value
$\mathrm{Vehicle}\;\mathrm{Radio}\;\mathrm{Range}\;(RR_{max}$)	150 m
Producer Vehicle	7 (unless specified otherwise)
Propagation Loss Model	*Nakagami* propagation loss model Range propagation loss model
Propagation Delay Model Highway	Constant speed propagation delay model
Producer vehicle placement Highway	Within the initial distance of 1.9 km Safety producers (1.1 km) (located at a distance of 1.5 km and 1.9 km)
Network density	130 vehicles (unless specified otherwise)
Road length	10 km
Replacement Policy	LRU
Caching Policy	LCE
TxPowerStart	5 (dbm)
TxPowerEnd	5 (dbm)
PEL	4 s
Speed Variation	20 (70–90 km/h) [30,31]
Consumer vehicle	8
Simulation time	500 s for each experiment
